# Determination of diurnal rhythm of salivary corticosterone concentration in the domestic rabbit (*Oryctolagus cuniculus* f. *domestica*) using a non-invasive method

**DOI:** 10.1038/s41598-023-33575-4

**Published:** 2023-04-19

**Authors:** Gabriela Kadlecova, Martina Volfova, Jan Chloupek, Monika Sebankova, Lucie Hostovska, Eva Voslarova, Vladimir Vecerek

**Affiliations:** 1 Department of Animal Protection and Welfare and Veterinary Public Health, Faculty of Veterinary Hygiene and Ecology, University of Veterinary Sciences Brno, Brno, Czech Republic; 2 Department of Pharmacology and Pharmacy, Faculty of Veterinary Medicine, University of Veterinary Sciences Brno, Brno, Czech Republic

**Keywords:** Biochemistry, Physiology

## Abstract

There are many advantages associated with the determination of the level of corticosterone in rabbits from saliva, since this is a non-invasive sample collection method that does not affect their welfare and provides a reliable reflection of the state of the animal at a given moment without the results being distorted as they may be, for example, when blood samples are taken. The aim of this study was to determine the diurnal rhythm in the concentration of corticosterone in the saliva of the domestic rabbit. Saliva samples were taken from six domestic rabbits five times during the daytime (at 6:00, 9:00, 12:00, 15:00 and 18:00) over the course of three consecutive days. The levels of corticosterone in the saliva of the individual rabbits displayed a diurnal rhythm during the course of the day, with a significant increase between 12:00 and 15:00 (p < 0.05). No statistically significant difference in the concentrations of corticosterone in the saliva of the individual rabbits was demonstrated. Although the basal value of corticosterone is not known in rabbits and is difficult to determine, the results of our study show the pattern of fluctuations in the concentration of corticosterone in the saliva of rabbits during the daytime.

## Introduction

The evaluation of stress in animals is a topical issue in the area of welfare, and non-invasive methods are continually being developed and perfected in an effort to develop procedures that are considerate to animals. Various biological materials, such as saliva, fur, urine, milk and faeces, serve as matrices for the analysis of metabolites of the glucocorticoid hormones cortisol and corticosterone^1–6^. The method used most frequently in the past for determination of the concentration of glucocorticoids was the sampling of the blood of the animal^7^. The collection of blood is, however, relatively stressful to the animal as it is associated with the pain of the needle insertion and with immobilization, and this may influence the results obtained. The limitations on the use of invasive sampling in very small animals, in which it is a highly risky procedure associated with a substantial loss of blood and the risk of subsequent mortality, in addition to its negative impact on the welfare of the individual, are also a problem. It is also not possible to repeat sampling on such animals and assess, for example, the hormonal state of the individual during ongoing illness or its fluctuation over the course of the day^8^. The secretion of glucocorticoid hormones is influenced by circadian rhythms, with fluctuations in the levels of the given hormones occurring during the course of 24-h cycles affected by both endogenous and exogenous factors (the alternation of day and night)^9^. The degree of the stress reaction of the individual cannot, therefore, be determined precisely from the concentrations measured in the blood, as they merely testify to the current state of the animal which is also affected by sampling itself^10^.

Non-invasive methods, in contrast, offer a number of advantages over invasive methods. They make it possible, for example, to monitor both short-term and long-term changes in the concentrations of glucocorticoids and their metabolites that are fundamental to longer-term studies that monitor, for example, the fluctuation in values during the individual seasons of the year, etc.^8^. The determination of the level of cortisol or corticosterone in the saliva is a method that is currently widely used in livestock animals and pets^2^. The results of the studies by Romero et al.^11^ indicate that the level of glucocorticoid hormones, and specifically cortisol, increases in the blood of a sampled animal 3 min or less after handling begins. In contrast, Kobelt et al.^2^ found that no increase in the levels of cortisol in the saliva occurred in animals within 4 min of handling beginning, which may indicate that that salivary concentration changes occur later than blood.

The domestic rabbit is a species frequently farmed intensively for the production of meat, as well as being used as a laboratory animal. Both on farms and research facilities rabbits may be exposed to a large number of stress factors that have a great effect on their quality of life^12,13^. Invasive methods such as blood sampling^14^ and non-invasive methods of determining the level of hormones in droppings^15^ have been used in the past for the evaluation of stress in rabbits, though only one study concerning sampling and determination of glucocorticoids from saliva is known^16^. It is essential during the evaluation of the levels of stress markers in rabbits from the saliva to be aware of the fact that, in contrast to the majority of mammals, they predominantly secrete corticosterone^17^ rather than cortisol. Corticosterone is also secreted to a larger extent by, for example, birds and rodents^11^. The advantages of the use of non-invasive methods consist of both consideration to the animal and the fact that the results obtained are not distorted by the stress caused by handling itself or by the pain accompanying sampling. ELISA Immunoassay kits can be used to determine corticosterone in the saliva^18^.

The aim of this study was to determine the fluctuation in the level of salivary corticosterone concentrations in the domestic rabbit during the daytime using a non-invasive method.

## Results

The median concentrations of corticosterone in the saliva of the six rabbits are given in Table 1. No significant differences were found between the individual rabbits in the median concentrations of corticosterone in the saliva at the individual sampling times (p > 0.05).

**Table 1:** Median concentrations of corticosterone (pg/ml) in the saliva of 6 rabbits at 6:00, 9:00, 12:00, 15:00, and 18:00.

Rabbit no	Time of day				
	6:00	9:00	12:00	15:00	18:00
	Median	Median	Median	Median	Median
1	617.53^a^	444.22^a^	110.73^a^	961.43^a^	553.92^a^
2	349.43^a^	246.91^a^	250.04^a^	750.77^a^	370.80^a^
3	492.55^a^	456.28^a^	650.35^a^	849.19^a^	555.10^a^
4	1058.86^a^	907.59^a^	815.09^a^	1304.92^a^	880.21^a^
5	928.14^a^	1035.02^a^	842.64^a^	1135.87^a^	859.94^a^
6	867.63^a^	986.23^a^	581.49^a^	1030.12^a^	618.27^a^

^a^The values in a column with the same superscript letter are not significantly different (p > 0.05).

**Figure 1:**
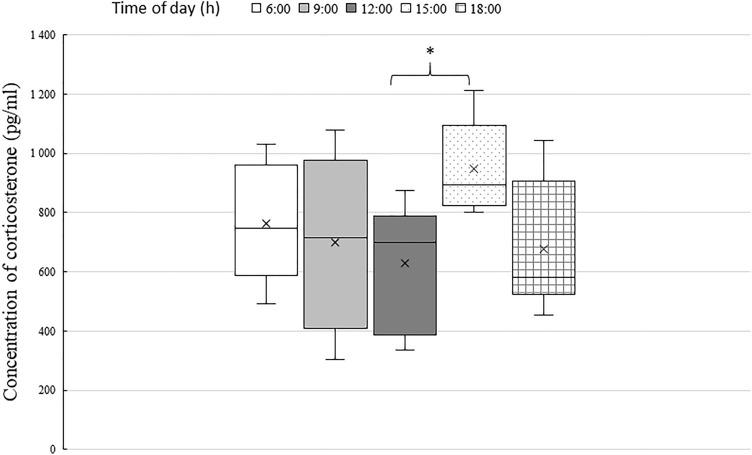
The daytime fluctuation of the salivary corticosterone concentration in six rabbits. Three replicate samples were taken and analysed at each point in time. ^a,b^The values with different superscript letters are significantly different (p < 0.05).

The diurnal rhythm in the levels of salivary corticosterone in the rabbits during the daytime is depicted in Fig. 1. A significant (p < 0.05) increase in the level of salivary corticosterone was found at 15:00 in comparison with the values determined earlier.

## Discussion

No differences in the concentration of corticosterone in the saliva were found between the six sampled rabbits at any of the sampling times. However, triplicate samples were taken at each point in time in order to determine the central tendency of the cortisol with minimal uncertainty rather than as a feature for determining differences between rabbits. A conclusive fluctuation in the level of salivary corticosterone was seen during the daytime. The diurnal fluctuation in the secretion of glucocorticoids is common, with a peak being recorded at different times in different species^19–22^. In the majority of diurnal animals, for example, a peak generally occurs towards the end of the period of darkness, while in primarily nocturnal animals a secretion peak occurs at the end of the period of light^21^. It is not entirely clear whether the rabbit is a diurnal or nocturnal animal^23–25^, though we recorded a significantly increased corticosterone levels in the saliva at 15:00 within the period monitored in our study (6:00–18:00). Similarly, Szeto et al.^26^ reported an increase in corticosterone in the evening hours in their study, specifically the level of corticosterone in the blood of rabbits attained its maximum values of 30.01 (± 2.62) ng/ml at around 18:00 h. In their study, unfortunately, the previous sampling took place at noon and there was no sampling at 15:00, so it is impossible to compare if there were also spikes in concentration between 12:00 and 15:00 h. The results of analysis of corticosterone in the blood and the saliva indicate, then, that secretion of corticosterone corresponds rather to the rabbit probably being an animal displaying nocturnal activity. However, external noise or feeding during the day may change its behavioural patterns to higher activity during the day^27^. For this reason, animals kept in laboratory conditions where they are fed mainly during the day when there is also more noise, could be stressed by sampling at night and the values would be elevated. In this case, saliva sampling only in 12-h cycles may be relevant.

In certain cases, no diurnal rhythm in glucocorticoid levels during the day need necessarily be recorded, particularly if the action of a strong stressor that may interfere with the circadian rhythm occurs^20^. For example, the levels of corticosterone measured in rabbit saliva in our study are very different from the levels measured in rabbits by Munari et al.^16^. Their study included evaluation of the levels of salivary corticosterone in rabbit does exposed to intensive reproductive rhythm, i.e. during a stressful period due to the enormous energy demand. The average values of corticosterone they measured in the saliva ranged from 1036 to 2929 ng/ml, while the average concentrations of salivary corticosterone measured in the rabbits sampled in our study ranged from 628 to 948 pg/ml. This can be explained by the fact that the rabbits in our study had a minimal stress load and were young males kept in conditions to which they were accustomed. In contrast, Munari et al.^16^ performed the sampling of saliva from older breeding does in various housing systems and at various stages of the reproductive cycle which may be associated with a certain degree of stress. To our knowledge, there are no other studies reporting levels of corticosterone in rabbit saliva, and further research is required to determine reference values of the salivary corticosterone concentration for various rabbit categories and the factors influencing it. Only the results of studies into the level of corticosterone in the blood of rabbits have been published to date^28^. Previous research indicates that only a fraction of free corticosterone from the blood is present in saliva^20^. Furthermore, it has been demonstrated, for example in rats^29^, that a small proportion of hormones may also be produced by the salivary glands. Cook et al.^30^ found the overall ratio of saliva to serum cortisol to be 9% in pigs. The concentration we determined in the saliva would confirm the assumption that the saliva contains around 10% of the concentration found in the blood^28,31^. In our study, neither blood samples were evaluated to confirm the correlation between corticosterone in blood and saliva, nor cortisol was administered to confirm the elevation of the salivary corticosterone after that. However, such research was carried out e.g. in mice^32^. Cook et al.^30^ found significant correlations between serum and salivary cortisol values following ACTH stimulation in pigs and even suggested salivary cortisol to be a better indicator of stress than cortisol measured in blood samples.

The secretion of corticosterone may also be influenced by other factors. In young domestic rabbits, for example, it is positively correlated with the growth of the animals at the time of milk intake^14^. Stress factors associated with the type of housing may have an effect on rabbits on intensive farms. The inadequate size of cages, for example, is associated with a rise in levels of stress hormones^33^. A rise in the level of corticosterone has been found in certain animals, such as rats, in connection with a restricted diet^34^, though in our study of rabbits in a laboratory environment with intake of feed ad libitum with no restrictions of any kind in the provision of feed it is not likely that a rise in the levels of corticosterone in the saliva in the afternoon hours was associated with a feeling of hunger. Monclús et al.^35^ state that certain stress situations cause a more significant increase in the level of stress hormones in males than in females. In our study, the determination of salivary corticosterone took place only in males at rest, and further research should, therefore, also focus on monitoring the level of corticosterone in the saliva of females or check for changes in both sexes resulting from the exposure to various stress factors.

## Conclusion

It is essential from the viewpoint of the welfare and health protection of animals reared for laboratory, farming or other purposes that we understand their response to stressors acting on them during their rearing and try to adapt their rearing conditions to correspond to the needs of the animals and to increase the level of their welfare. Evaluation of the corticosterone concentrations in the saliva of rabbits may serve this purpose. To date, there is limited knowledge of the values of corticosterone in the saliva of rabbits. In this study, it has been shown that the concentrations of corticosterone in rabbit saliva fluctuate during the daytime even without the action of any evident stress, and consideration must be given to this fact during the interpretation of the results, particularly when this method is used to evaluate stress. Further research will, however, be necessary for the determination of a reference range and to judge the extent to which age, sex and possible other factors play a role.

## Materials and methods

### Animals

For the purposes of the study, six adult rabbits of the HYLA hybrid were selected at random from a laboratory farm (Farma Kočárovi s.r.o., Jaroměřice nad Rokytnou, Czech Republic). These were males of a weight of around 3 kg that were placed in stainless-steel hutches with a plastic grate and platform in premises with controlled environmental conditions including a 12-h light regime. The temperature was maintained within a range of 17–23 °C and the humidity at 45–60%. Water was provided ad libitum, and the rabbits were also fed ad libitum with the complete feed mix Biostan KBO (Bikron, Blučina, Czech Republic). The rabbits were in visual, olfactory and vocal contact with one another and were accustomed to human handling during regular weighing. No other scientific or experimental interventions were performed on these animals during the period of saliva sampling.

### Sample collection

A series of samplings was performed over the course of three consecutive days. Each day, sampling was performed five times during the daytime at 6:00, 9:00, 12:00, 15:00 and 18:00. A total of 15 samples were taken from each rabbit. Saliva samples were taken from the mouth with a Salivette sampling set (Sarstedt, Nümbrecht, Germany). A sampling swap was inserted into the corner of the rabbit’s mouth with the use of a haemostat and was subsequently chewed by the animal for 60–90 s. The Salivette sets were centrifuged immediately after sampling (6000 rpm/15 min). The saliva samples were then placed in Eppendorf test tubes, frozen and stored at − 80 °C. The amount of saliva taken from the individual rabbits varied within a range of 0.02–0.5 ml.

### Corticosterone analysis

A Corticosterone Competitive ELISA Kit (ThermoFisher Scientific, Czech Republic) designed for the determination of corticosterone with a sensitivity of 18.6 pg/ml and detection limits of 78.125–10,000 pg/ml was used for the detection of corticosterone in the saliva of the rabbits. Evaluation was performed on a calorimetric sensor at a wavelength of 450 nm and the results read on the curve created by the ELISA calculator of the Arigo software (www.arigobio.com).

### Statistical analysis

The values recorded for each rabbit at a certain time were calculated as the average of the values of the three samples taken at the same time (at 6:00, 9:00, 12:00, 15:00 and 18:00) on the three consecutive days for the purposes of evaluation. The results were analyzed using the statistical package Unistat 5.6. (Unistat Ltd., London, England). The normality of data was checked using the Shapiro–Wilk test. As the data was not distributed normally, the nonparametric Friedman ANOVA test with a non-parametric multiple comparison was used to determine the differences between the values measured in the rabbits at the individual studied times and for the purposes of evaluating the fluctuation in the levels of corticosterone in each rabbit during the day. A value of p < 0.05 was determined as statistically significant in statistical tests.

### Ethical approval

This study was carried out in strict accordance with the Directive 2010/63/EU of the European Parliament and of the Council of 22 September 2010 on the protection of animals used for scientific purposes and Czech national legislation, that is, Act No. 246/1992 Coll., on the protection of animals against cruelty, as amended. All procedures were of routine veterinary practice and informed consent has been kept. Animal Experimentation Ethics Committee of the University of Veterinary Sciences Brno was informed of all procedures and agreed to carry out the aforementioned clinical study. All procedures were carried out in compliance with the ARRIVE guidelines (https://arriveguidelines.org).

## Data Availability

Kadlecová, Gabriela (2022): Saliva corticosterone in the Domestic Rabbit (Oryctolagus cuniculus f. domestica). figshare. Dataset. https://doi.org/10.6084/m9.figshare.21455910.v3.
